# Ginkgolide B Reduces LOX-1 Expression by Inhibiting Akt Phosphorylation and Increasing Sirt1 Expression in Oxidized LDL-Stimulated Human Umbilical Vein Endothelial Cells

**DOI:** 10.1371/journal.pone.0074769

**Published:** 2013-09-17

**Authors:** Lina Ma, Xueqing Liu, Yanyang Zhao, Beidong Chen, Xingguang Li, Ruomei Qi

**Affiliations:** 1 The Key Laboratory of Geriatrics, Beijing Hospital & Beijing Institute of Geriatrics, Ministry of Health, Beijing, P. R. China; 2 Beijing University of Chinese Medicine, Beijing, P. R. China; University of Kansas Medical Center, United States of America

## Abstract

Oxidized low-density lipoprotein (ox-LDL) is an important risk factor in the development of atherosclerosis. LOX-1, a lectin-like receptor for ox-LDL, is present primarily on endothelial cells and upregulated by ox-LDL, tumor necrosis factor a, shear stress, and cytokines in atherosclerosis. Recent studies demonstrated that ginkgolide B, a platelet-activating factor receptor antagonist, has antiinflammatory and antioxidant effects on endothelial and nerve cells. The present study investigated the effects of ginkgolide B on LOX-1 expression and the possible mechanism of action. Our results showed that ginkgolide B inhibited LOX-1 and intercellular cell adhesion molecule-1 (ICAM-1) expression in ox-LDL-stimulated endothelial cells through a mechanism associated with the attenuation of Akt activation. Similar data were obtained by silencing Akt and LY294002. We also evaluated Sirt1 and nuclear factor erythroid 2-related factor 2 (Nrf2) expression. These molecules play a protective role in endothelial cell injury. The results showed that ginkgolide B increased Sirt1 expression in ox-LDL-treated cells. The inhibitory effects of ginkgolide B on LOX-1 and ICAM-1 expression were reduced in Sirt1 siRNA-transfected cells. Nrf2 expression was increased in ox-LDL-treated cells, and ginkgolide B downregulated Nrf2 expression. These results suggest that ginkgolide B reduces Nrf2 expression by inhibiting LOX-1 expression, consequently reducing oxidative stress injury in ox-LDL-stimulated cells. Altogether, these results indicate that the protective effect of ginkgolide B on endothelial cells may be attributable to a decrease in LOX-1 expression and an increase in Sirt1 expression in ox-LDL-stimulated endothelial cells, the mechanism of which is linked to the inhibition of Akt activation. Ginkgolide B may be a multiple-target drug that exerts protective effects in ox-LDL-treated human umbilical vein endothelial cells.

## Introduction

Oxidized low-density lipoprotein (ox-LDL) is a crucial factor in triggering the development of atherosclerosis. In endothelial cells, ox-LDL is taken up by lectin-like oxidized low-density lipoprotein receptor-1 (LOX-1) and then stimulates the intracellular inflammatory response [1]–[4]. LOX-1 is a type II membrane glycoprotein, and its expression is regulated by numerous factors, such as tumor necrosis factor a (TNF-a), shear stress, and ox-LDL [5]–[7]. These factors are all related to inflammation in atherosclerosis. Previous studies detected LOX-1 overexpression in atherosclerotic plaque and injured endothelial cells [8]–[10]. Therefore, the inhibition of LOX-1 expression is considered a valuable therapeutic strategy against atherosclerosis.

Ginkgolide B is an inhibitor of platelet-activating factor (PAF), which can inhibit platelet function. Our previous studies showed that ginkgolide B inhibited inflammatory protein expression induced by ox-LDL in human umbilical vein endothelial cells (HUVECs), such as intercellular adhesion molecule-1 (ICAM-1) and monocyte chemotactic protein-1 (MCP-1) expression, by inhibiting nuclear factor-kB (NF-kB) activation and reducing Nox4 expression in ox-LDL-treated endothelial cells [11], [12]. However, whether ginkgolide B influences LOX-1 expression in HUVECs has not yet been determined.

Phosphoinositide 3-kinases (PI3Ks) comprise a family of lipid kinases. The PI3K family has three distinct subgroups: class I (A and B), class II, and class III. PI3K kinase activation generates lipid second messengers by phosphorylating the head group of phosphoinositisides at the 3′ end. The effects of PI3K are transmitted through these lipid products, which bind to and regulate downstream protein effectors [13]. Protein kinase B (PKB/Akt) is a serine/threonine kinase and effector of PI3K. The PI3K/Akt pathway is involved in the regulation of numerous cell functions. Whether PI3K/Akt pathway activation is involved in ox-LDL-induced LOX-1 expression has not yet been determined. Therefore, the present study investigated whether ginkgolide B affects Akt phosphorylation in ox-LDL-stimulated endothelial cells.

Sirtuin 1 (Sirt1) is a NAD^+^-dependent lysine deacetylase that plays multiple roles in chromatin remodeling, cell ageing, organism longevity, energy metabolism, genomic stability, stress responses, and apoptosis [14]. Sirt1 is a nicotinamide adenine dinucleotide-dependent class III histone deacetylase that can downregulate the expression of various proinflammatory cytokines by inhibiting the NF-kB pathway [15]–[18]. Recent studies showed that Sirt1 has protective effects on macrophages and endothelial cells and in thrombosis [19]. Stein *et*. reported that Sirt1 reduced the uptake of ox-LDL by diminishing the expression of LOX-1 by suppressing the NF-kB signaling pathway [20].

Nuclear factor erythroid 2-related factor 2 (Nrf2) is a redox-sensitive transcription factor that plays a key role in cellular antioxidant defense. Under normal conditions, Nrf2 is sequestered in the cytoplasm by an inhibitory protein, Keap-1. In the presence of oxidative stress, Nrf2 is rapidly degraded by the proteasome system, enters the nucleus, binds to antioxidant response element (ARE), and upregulates multiple antioxidant and detoxifying genes [21]. Accumulating evidence has demonstrated that Nrf2 is activated in various oxidative stress-mediated diseases, such as neurodegenerative disease, cardiovascular disease, diabetes, and auto-immune disease [22]–[23]. Our previous studies showed that ginkgolide B can reduce the ox-LDL-induced generation of reactive oxygen species in endothelial cells [12]. In the present study, we investigated whether ginkgolide B influences Nrf2. We examined the effects of ginkgolide B on LOX-1 expression and subsequent protective effects in HUVECs.

## Materials and Methods

### Ethics statement

According to the Declaration of Helsinki, umbilical cords were donated by cesarean section patients, from whom we received written informed consent. The study was approved by the ethics committee of the Beijing Institute of Geriatrics, Ministry of Health.

### Materials

Antibodies against the Nrf2, and b-actin were purchased from Santa Cruz Biotechnology (Santa Cruz, CA, USA). Anti-LOX-1 antibody was purchased from Abcam (Cambridge, UK). Anti-Sirt1 antibody was purchased from Cell Signaling Technology (Danvers, MA, USA). Pyrrolidine dithiocarbamate, phenylmethylsulfonyl fluoride, and leupeptin were purchased from Sigma (St. Louis, MO, USA). Horseradish peroxidase-conjugated anti-mouse and anti-rabbit immunoglobulin G antibodies were purchased from Beijing Zhongshan Golden Bridge Biotechnology (Beijing, China). Ginkgolide B (95% purity) was purchased from Daguanyuan Company (Xuzhou, Jiangsu, China).

### Preparation of LDL and ox-LDL

Human LDL was isolated from freshly obtained serum by sequential ultracentrifugation. Low-density lipoprotein was oxidized with 5 mM CuSO_4_ for 16 h at 37°C, and oxidation was stopped by the addition of 20 mM ethylenediaminetetraacetic acid (EDTA). Oxidation was confirmed using the thiobarbituric acid-reactive substance assay [24]. The ox-LDL preparation was filtered through 0.22 mm filters and stored at 4°C. The protein concentration of ox-LDL was determined by a spectrophotometer at a wavelength of 280 nm (ultraviolet-visible record spectrophotometer, Shimadzu, Japan).

### Cell culture

Human umbilical cords were obtained from healthy donors who provided written informed consent. Human umbilical vascular endothelial cells were isolated from the fresh umbilical vein, and the isolated cells were cultured in Dulbecco's modified Eagle's medium (DMEM) that contained 10% fetal bovine serum, 2 mM glutamine, and antibiomycin (10 mM penicillin G and 10 mM streptomycin) at 37°C in a humidified 5% CO_2_ atmosphere. Human umbilical vascular endothelial cells at passages 3–5 were used in the present study.

### Cell preparation and ox-LDL treatment

The cells were pretreated with various doses of ginkgolide B for 1 h and then exposed to ox-LDL for 4 h. The cells were then lysed by dissolving in lysis buffer (100 mM Tris/HCl, pH 7.2, 50 mM NaCl, 5 mM EDTA, 2 mM vanadata, 1 mM phenylmethylsulfonyl fluoride, and 100 mg/ml leupeptin). The samples were then sonicated and centrifuged at 15,000×*g* for 5 min. The lysates were subjected to Western blot using specific antibodies.

### Western blot

Cell lysates were analyzed by sodium dodecyl sulfate-polyacrylamide gel electrophoresis and electrotransferred to polyvinylidene fluoride membranes. The membranes were blocked with 1% bovine serum albumin and then incubated with specific antibodies. After three washes in Tris phosphate-buffered saline (TPBS) that contained 0.5% Tween 20 in PBS, the membranes were incubated with horseradish peroxidase-conjugated secondary antibodies in TPBS. The bands were detected by chemiluminescent detection reagents. Blot densitometry was then performed, and the bands were analyzed using a Gene Genius Bio Imaging System.

### Quantitative RT-PCR analysis

Total RNA was extracted from primary HUVECs using Trizol reagent (Invitrogen) and subjected to reverse transcription. Real-time polymerase chain reaction (RT-PCR) was performed using SYBR Premix Ex Taq mix (Takara) according to the manufacturer's instructions and run on an iQ5 Multicolor Real-time PCR Detection System (Bio-Rad, Hercules, CA, USA). Thermal reaction cycles of 95°C for 30 s and 45 repetitions of 95°C for 5 s at 60°C for 20 s were used. Real-time PCR data were analyzed using the ΔΔC_T_ method, normalizing the C_T_ values of the indicated gene to the C_T_ values of glyceraldehyde-3-phosphate dehydrogenase (GAPDH) relative to a control sample. The sequences for the primer pairs were the following: Sirt1 (sense, 5′-CCT GAC TTC AGA TCA AGA GAC GGT-3′; antisense, 5′-CTG ATT AAA AAT GTC TCC ACG AAC AG-3′), Nrf2 (sense, 5′-TTC AGC CAG CCC AGC ACA TC-3′; antisense, 5′-CGT AGC CGA AGA AAC CTC ATT GTC-3′), GAPDH (sense, 5′-CAA CAG CCT CAA GAT CAT CAG CA-3′; antisense, 5′-TGG CAT GGT CTG TGG TCA TGA GT-3′). The ratios of the amplified targets were compared with the amplified internal control GADPH. Quantitative densitometry analysis was performed using Quantity One software (Bio-Rad).

### Sirt1 and Akt small-interfering RNA

HUVECs were cotransfected with effectence transfection reagent (QUAGEN) that carried Sirt1-selective small-interfering RNA (siRNA) and Akt-selective siRNA (#6211, Cell Signaling Technology, Danvers, MA, US). The Sirt1 siRNA sequences were 5′-ACUUUGCUGUAACCCUGUA(dTdT)-3′ and 5′-UACAGGGUUACAGCAAAGU((dTdT)-3′ [25]. Transfected cells were cultured for 72 h to silence Sirt1 and Akt, respectively. Immunoblotting was performed to examine the efficiency of protein knockdown.

### Statistical analysis

The data are expressed as mean ± SEM. The statistical analyses were performed using independent *t*-tests and analysis of variance (ANOVA), followed by the Tukey *post hoc* test. The results were considered significant at *p*<0.05.

## Results

### Effect of ginkgolide B on LOX-1 expression

**Figure 1:**
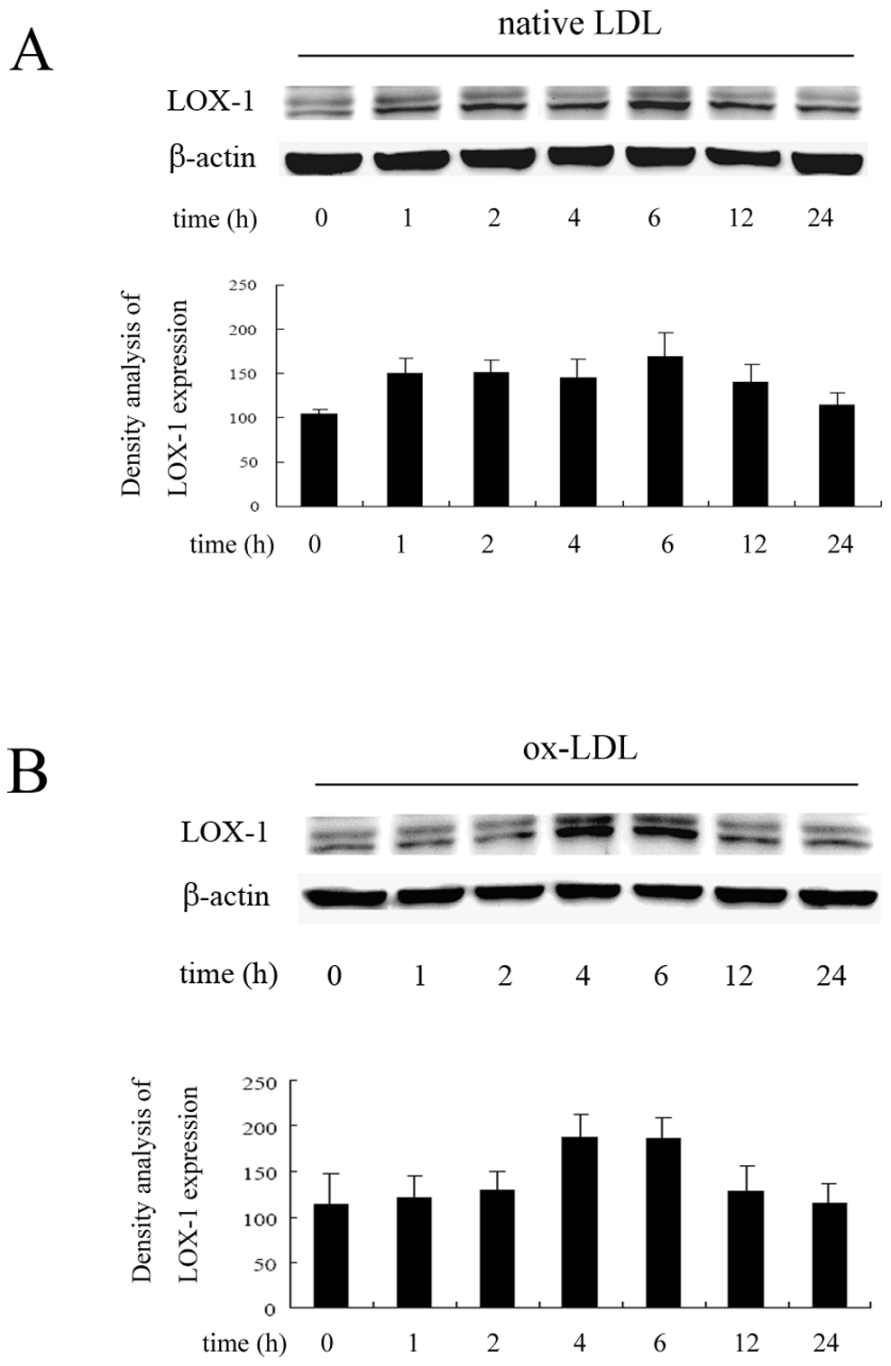
LOX-1 expression induced by native LDL or ox-LDL at different time points. (A) Native LDL-induced LOX-1 expression. (B) ox-LDL-induced LOX-1 expression. The results were obtained from three independent experiments.

**Figure 2:**
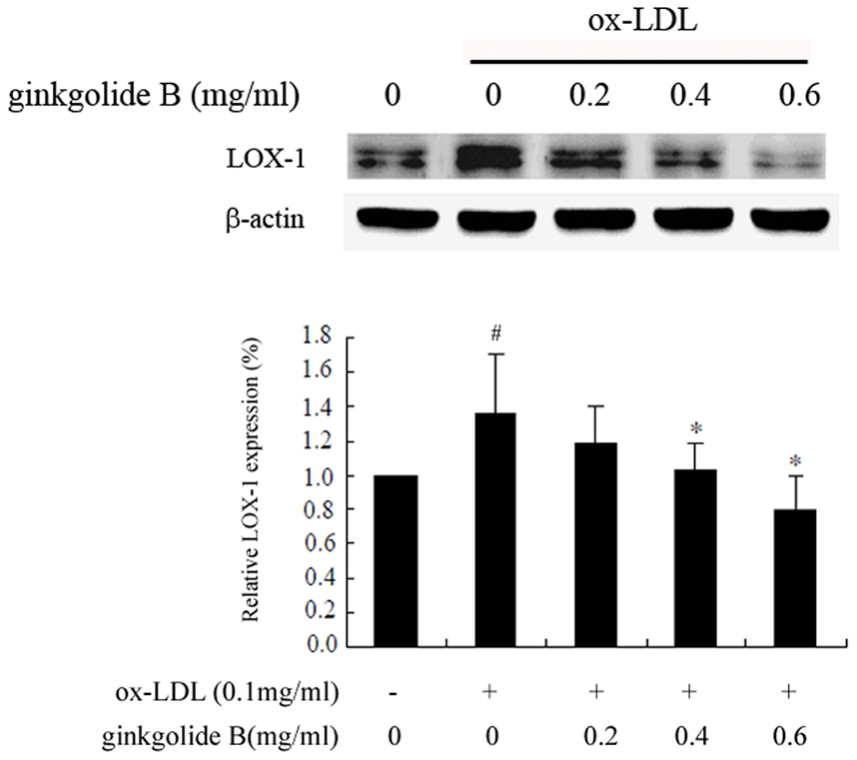
Ginkgolide B inhibits LOX-1 in ox-LDL-stimulated endothelial cells. The results were obtained from five independent experiments. ^#^ *p*<0.05, compared with control; *p<0.05, compared with ox-LDL-stimulated cells.

Ox-LDL cell entry is mediated by LOX-1, stimulates cell activation, and leads to an inflammatory response in endothelial cells. In the present study, we first observed the effect of ginkgolide B on LOX-1 expression. Native LDL and ox-LDL were used to determine whether both exhibit differences in HUVECs. As shown in Fig. 1, the cells were treated with ox-LDL or native LDL for various durations. LOX-1 expression was obvious increased by ox-LDL treatment for 4 and 6 h. We then investigated the effect of ginkgolide B on LOX-1 expression in cells treated with ox-LDL for 4 h. As shown in Fig. 2, LOX-1 expression increased by 36% in ox-LDL-treated cells, and 0.4 and 0.6 mg/ml ginkgolide B dose-dependently attenuated LOX-1 expression. The results suggest that ginkgolide B may reduce ox-LDL entry into cells by inhibiting LOX-1 expression.

### Effect of ginkgolide B on Akt phosphorylation

**Figure 3:**
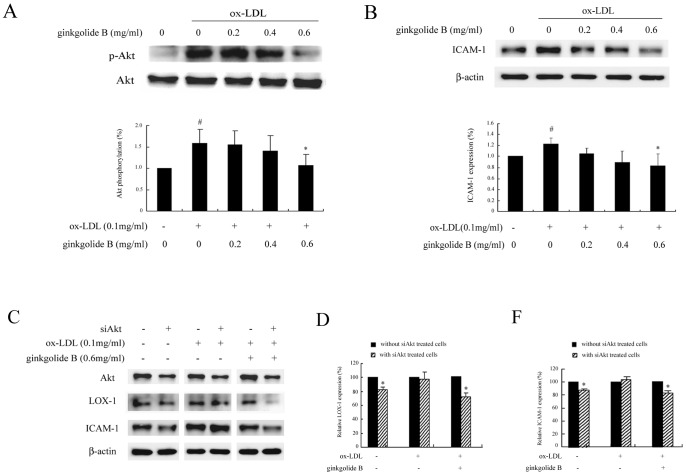
Ginkgolide B inhibits Akt phosphorylation and ICAM-1 expression in ox-LDL-stimulated endothelial cells. (A) Ginkgolide B inhibited Akt phosphorylation. ^#^ *p*<0.05, compared with control; *p<0.05, compared with ox-LDL-stimulated cells. (B) Ginkgolide B suppressed ICAM-1 expression. ^#^ *p*<0.05, compared with control; *p<0.05, compared with ox-LDL-stimulated cells. (C) LOX-1 and ICAM-1 expression in Akt1 siRNA -transfected and -nontransfected cells. (D) Analysis of LOX-1 expression with Akt siRNA transfection. The results were obtained from three independent experiments. *p<0.05, difference between Akt siRNA-transfected and -nontransfected cells. (F) Analysis of ICAM-1 expression with Akt siRNA transfection. *p<0.05, difference between Akt siRNA-transfected and -nontransfected cells.

Our previous data indicated that ginkgolide B inhibited Akt phosphorylation in activated platelets. Therefore, we sought to determine whether ginkgolide B affects Akt phosphorylation in ox-LDL-treated HUVECs. We determined the level of Akt phosphorylation induced by various concentrations of ox-LDL, and 0.1 mg/ml ox-LDL induced stronger Akt phosphorylation (data not shown). Based on these results, we used the 0.1 mg/ml concentration of ox-LDL in the present study. As shown in Fig. 3A, Akt phosphorylation increased by 53% in ox-LDL-stimulated cells, and ginkgolide B dose-dependently decreased Akt phosphorylation. At a dose of 0.6 mg/ml, ginkgolide B almost completely inhibited Akt phosphorylation. We then evaluated the effect of ginkgolide B on ICAM-1 expression. As shown in Fig. 3B, ICAM-1 expression increased by 22% in ox-LDL-treated cells, and 0.6 mg/ml ginkgolide B fully suppressed ICAM-1 expression.

**Figure 4:**
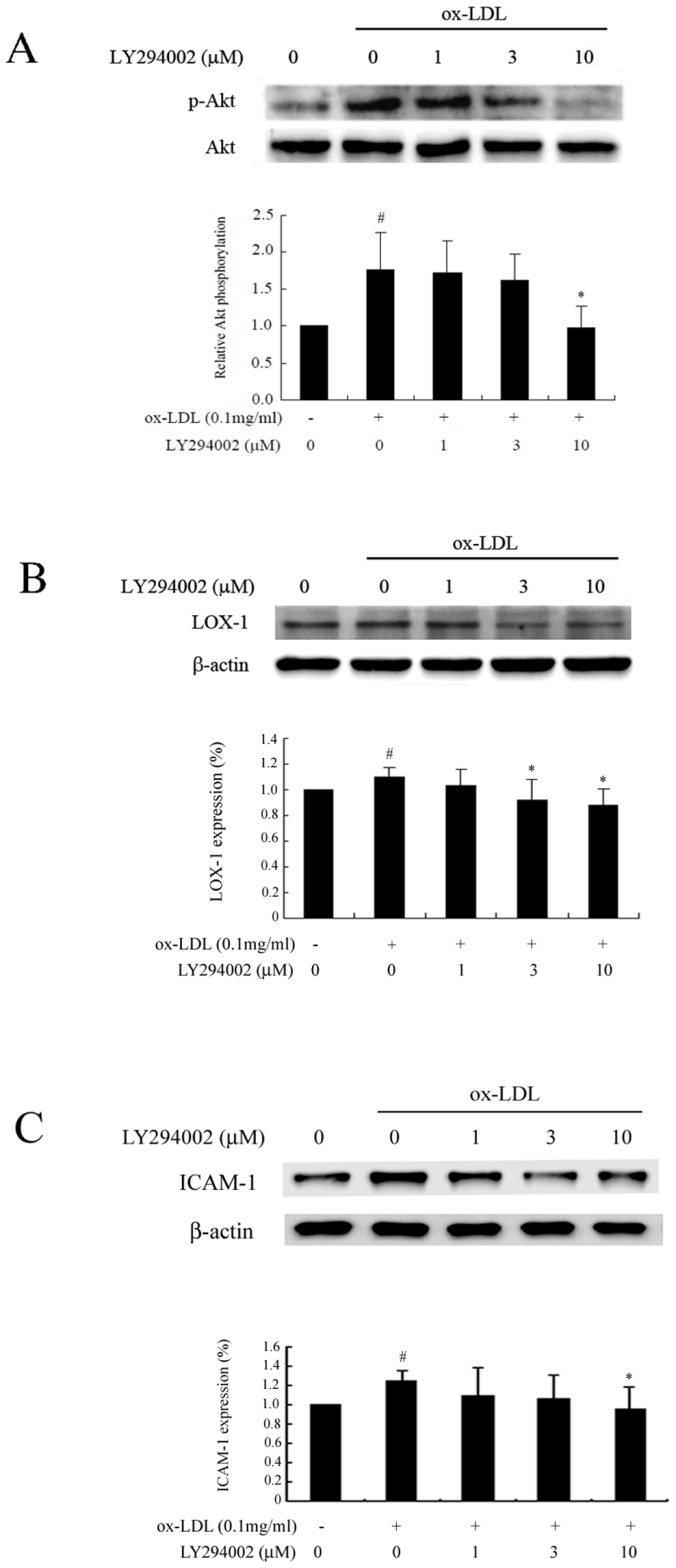
LY294002 inhibits Akt phosphorylation and LOX-1 and ICAM-1 expression in ox-LDL-stimulated endothelial cells. (A) LY294002 suppressed Akt phosphorylation. (B) LY294002 diminished LOX-1 expression. (C) LY294002 reduced ICAM-1 expression. The data were obtained from five independent experiments. ^#^ *p*<0.05, compared with control; *p<0.05, compared with ox-LDL-stimulated cells.

To further confirm the effect of Akt activation on LOX-1 and ICAM-1 expression, we used Akt siRNA to inhibit Akt function. As shown in Fig. 3C, Akt expression was downregulated in Akt siRNA-transfected cells. LOX-1 and ICAM-1 expression was significantly reduced in untreated cells, whereas no measurable effect was detected in cells treated with ox-LDL alone. In contrast, LOX-1 and ICAM-1 expression was markedly decreased by ginkgolide B in ox-LDL-stimulated and Akt siRNA-transfected cells. This suggests that the ginkgolide B-induced inhibition of LOX-1 expression occurs through Akt signaling. We then used LY294002, a specific inhibitor of PI3K that can inhibit Akt phosphorylation. As shown in Fig. 4, 10 mM LY294002 potently suppressed Akt phosphorylation and simultaneously reduced LOX-1 expression in ox-LDL-stimulated endothelial cells. Moreover, ICAM-1 expression was attenuated by 10 mM LY294402 treatment in ox-LDL-stimulated cells. These results further indicate that LOX-1 expression is regulated by the Akt pathway, and the ginkgolide B-induced inhibition of LOX-1 expression may be attributable to the attenuation of Akt phosphorylation.

### Effect of ginkgolide B on p38MAPK phosphorylation

**Figure 5:**
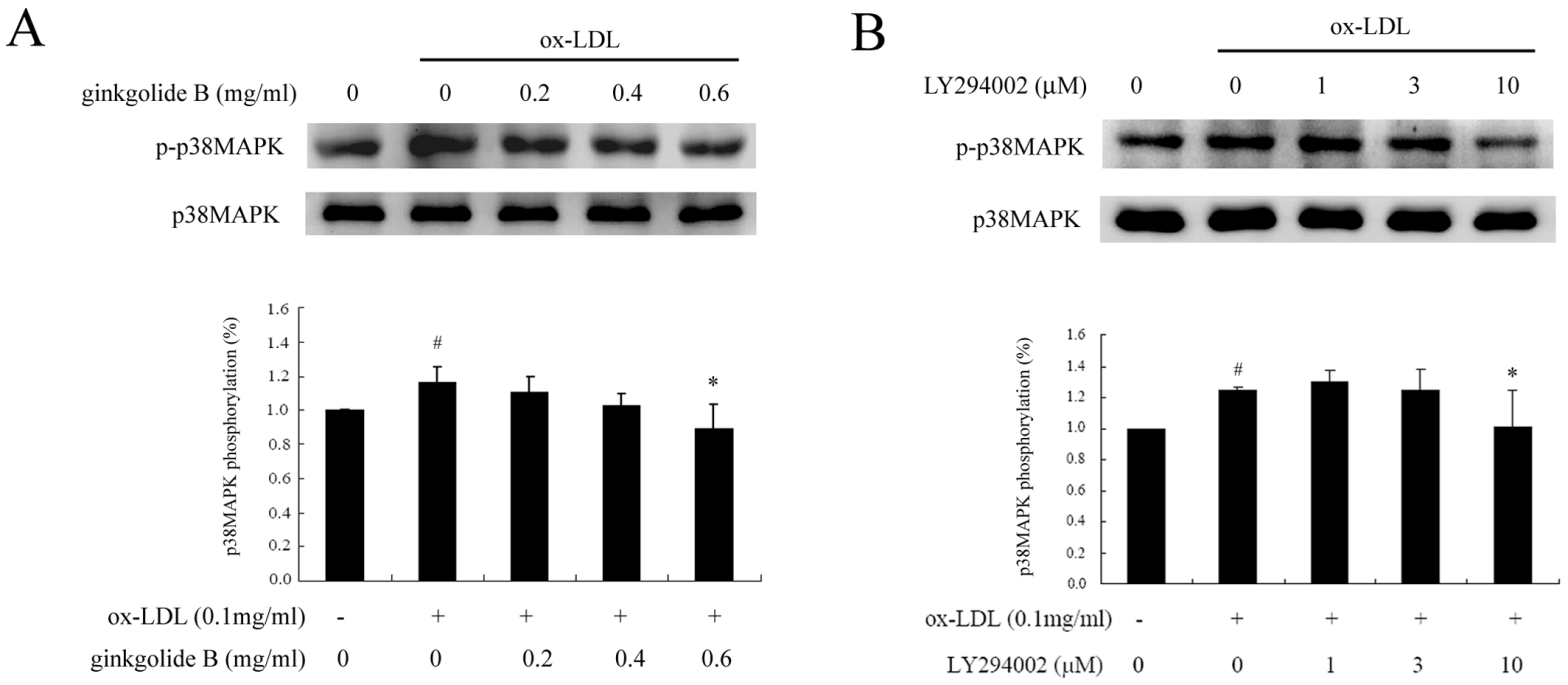
Ginkgolide B and LY294002 inhibit p38MAPK phosphorylation. (A) Ginkgolide B inhibited p38MAPK phosphorylation. (B) LY294002 reduced p38MAPK phosphorylation. ^#^ *p*<0.05, compared with control; *p<.05, compared with ox-LDL-stimulated cells. The data were obtained from five independent experiments.

p38-mitogen-activated protein kinase (p38MAPK) plays a critical role in ox-LDL-simulated endothelial cell injury. In the present study, we investigated whether the ginkgolide B-induced inhibition of LOX-1 expression is associated with p38MAPK activation. As shown in Fig. 5, ox-LDL increased p38MAPK phosphorylation by 16%, and 0.6 mg/ml ginkgolide B significantly attenuated p38MAPK phosphorylation in ox-LDL-treated cells. Similarly, 10 mM LY294002 fully abolished p38MAPK phosphorylation. These results suggest that p38MAPK might be a downstream molecule, and its activation might be regulated by Akt signaling in ox-LDL-stimulated cells.

### Effect of ginkgolide B on Sirt1 expression

**Figure 6:**
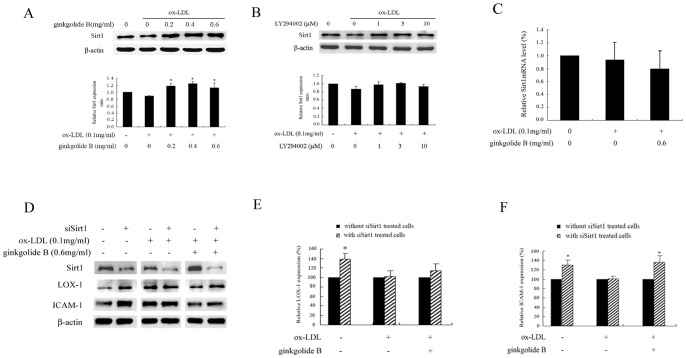
Ginkgolide B increases Sirt1 expression in ox-LDL-stimulated endothelial cells. (A) Ginkgolide B increased Sirt1 expression. ^#^ *p*<0.05, compared with control; *p<0.05, compared with ox-LDL-stimulated cells. (B) LY294002 had no effect on Sirt1 expression. (C) Quantitative analysis of the level of Sirt2 mRNA by RT-PCR. GADPH was used as an internal reference in the reactive system. (D) Sirt1 siRNA increased LOX-1 and ICAM-1 expression. (E) Analysis of LOX-1 expression in Sirt1 siRNA-transfected cells. *p<0.05, difference between Sirt1 siRNA-transfected and -nontransfected cells. (F) Analysis of ICAM-1 expression in Sirt1 siRNA-transfected cells. *p<0.05, difference between Sirt1 siRNA-transfected and -nontransfected cells. The data were obtained from three independent experiments.

Accumulating evidence indicates that Sirt1 plays an important role in cardiovascular cell function in aging and disease. A recent study showed that Sirt1 expression was decreased in aged and atherosclerotic vessels *in vivo* [26]. We investigated whether the protective effect of ginkgolide B is linked to Sirt1 in ox-LDL-induced endothelial cell injury. As shown in Fig. 6A, Sirt1 exhibited a trend toward decreased expression in ox-LDL-treated endothelial cells, whereas Sirt1 expression increased in cells treated with ginkgolide B, even at a low concentration (0.2 mg/ml). We investigated whether Akt activation affects Sirt1 expression in ox-LDL-stimulated cells. As shown in Fig. 6B, Sirt1 expression did not significantly change in LY294002-treated cells. Then the mRNA abundance of Sirt1 was also determined. We performed RT-PCR to measure the level of Sirt1 mRNA. As shown in Fig. 6C, Sirt1 mRNA expression was depressed in ox-LDL-treated cells, and ginkgolide B had no effect on Sirt1 mRNA expression. This indicates that ginkgolide B appeared to modulate Sirt1 expression at the posttranscriptional level. These results suggest that the protective effect of ginkgolide B might be linked to upregulated Sirt1 expression, but this action occurs independently of the PI3K/Akt pathway.

We further confirmed the action of Sirt1 on LOX-1 expression and inflammatory protein expression induced by ox-LDL using Sirt1 siRNA. As shown in Fig. 6D, Sirt1 expression was markedly reduced in Sirt1 siRNA-transfected cells, whereas LOX-1 and ICAM-1 expression was increased under baseline conditions. No significant difference was found between Sirt1 siRNA-transfected and -nontransfected ox-LDL-stimulated cells. Moreover, ginkgolide B attenuated LOX-1 and ICAM-1 expression induced by ox-LDL in negative control cells, but the inhibitory capacity decreased in Sirt1 siRNA-transfected cells. These results indicate that the protective effect of ginkgolide B on endothelial cells at least partially depends on Sirt1.

### Effect of ginkgolide B on Nrf2 expression

**Figure 7:**
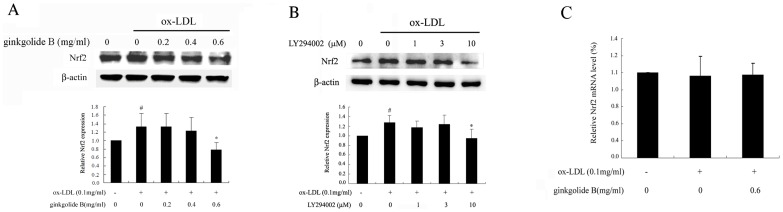
Ginkgolide B decreases Nrf2 expression without reducing the level of Nrf2 mRNA in ox-LDL-stimulated endothelial cells. (A) Ginkgolide B downregulated Nrf2 expression. (B) LY294002 recovered Nrf2 expression. The data were obtained from five independent experiments. ^#^ *p*<0.05, compared with control; *p<0.05, compared with ox-LDL-stimulated cells. (C) Quantitative analysis of the level of Nrf2 mRNA by RT-PCR. GADPH was used as an internal reference in the reactive system. The data were obtained from three independent experiments.

The Nrf2 pathway has been shown to restore redox homeostasis by increasing the antioxidant/electrophilic response element-mediated (ARE/EpRE) expression of phase II and antioxidant enzymes, including nicotinamide adenine dinucleotide phosphate-oxidase (NADPH), heme oxygenase-1 (HO-1), and the g-glutamate cysteine ligase catalytic subunit (GCLC) [27], [28]. Our previous studies found that ginkgolide B possessed antioxidant effects by inhibiting Nox4 mRNA expression in ox-LDL-treated endothelial cells, which is a subunit of the NADPH family. In the present study, we further examined whether ginkgolide B affects Nrf2 activation. As shown in Fig. 7A, Nrf2 expression increased by 33% in ox-LDL-treated cells, and Nrf2 expression was restored to baseline levels by ginkgolide B treatment in ox-LDL-stimulated cells. We examined the effect of LY294002 on Nrf2 expression. As shown in Fig. 7B, 10 mM LY294002 recovered Nrf2 expression in ox-LDL-treated cells. The level of Nrf2 mRNA was estimated. As shown in Fig. 7C, no significant changes in Nrf2 mRNA expression were induced by ginkgolide B treatment. These results suggest that the ginkgolide B-induced downregulation of Nrf2 expression might be linked to the inhibition of LOX-1 expression, thus leading to a reduced stress response in endothelial cells.

## Discussion

Atherosclerosis is characterized by the deposition of lipids and inflammatory cell infiltration in the vascular wall. Oxidized LDL plays a key role in endothelial cell injury. LOX-1 is an important mediator of endothelial cell insult in atherosclerosis. In the present study, we showed that ginkgolide B downregulated the expression of LOX-1 and inflammatory protein ICAM-1 expression in ox-LDL-stimulated endothelial cells. A previous study reported that ox-LDL, through its receptor LOX-1, triggered the CD40/CD40L signaling pathway, which activated the inflammatory response in human coronary artery endothelial cells [29]. We recently reported that ginkgolide B decreased CD40L expression on plaque in apolipoprotein E (ApoE) gene knockout mice [30]. In the present study, we further identified a novel target, in which ginkgolide B attenuated LOX-1 expression induced by ox-LDL in endothelial cells. Moreover, we assessed the regulatory mechanism of LOX-1 expression in ox-LDL-stimulated endothelial cells. ICAM-1 is an adhesion protein that is highly expressed in ox-LDL-stimulated HUVECs. Our results indicated that the ginkgolide B-induced suppression of LOX-1 and ICAM-1 expression is linked to the inhibition of Akt and p38MAPK phosphorylation. Similar results were observed in LY294002-treated endothelial cells. However, Akt silencing lacked such an inhibitory effect on LOX-1 and ICAM-1 expression in ox-LDL-stimulated cells. These results suggest that the protective effect of ginkgolide B on endothelial cells may occur through multiple targets.

Numerous studies have demonstrated that Sirt1 improves endothelial function, reduces inflammatory protein expression, and stabilizes plaques in vascular smooth muscle cells [31], [32]. Sirt1 decreases macrophage foam formation by inhibiting cholesterol uptake, suggesting that Sirt1 plays a protective role in atherosclerosis [33]. Therefore, we investigated the effect of ginkgolide B on Sirt1 expression. The results showed that ginkgolide B upregulated Sirt1 expression in ox-LDL-treated endothelial cells. The Sirt1 silencing experiment showed that Sirt1 siRNA increased LOX-1 and ICAM-1 expression under baseline conditions. The inhibitory effect of ginkgolide B on LOX-1 and ICAM-1 expression decreased in Sirt1 siRNA-transfected cells. However, LOX-1 and ICAM-1 expression in ox-LDL-stimulated cells did not change in Sirt1 siRNA-transfected cells. These results suggest that the blockade of ox-LDL-induced endothelial cell injury by ginkgolide B partially depends on Sirt1 signaling. Notably, Akt siRNA and Sirt1 siRNA transfection did not change LOX-1 or ICAM-1 expression in ox-LDL-treated cells, suggesting that inflammatory protein expression may be regulated by several signaling mechanisms in ox-LDL-injured cells.

Nrf2 has been reported to regulate a wide array of AREs and antioxidant proteins, such as NADPH. Nrf2 is a basic leucine zipper transcription factor that was originally identified as a binding protein of the locus control region of the b-globin gene [34], [35]. Many studies showed that the Nrf2-ARE pathway plays a critical role in cellular protection [36]-[38]. In the present study, we determined the effect of ginkgolide B on Nrf2 expression. Nrf2 is an inducible nuclear transcriptional factor that is highly expressed under stress conditions. Our results showed that ox-LDL stimulation enhanced Nrf2 expression, an effect that was blocked by ginkgolide B. This effect may be attributable to the ginkgolide B-induced inhibition of LOX-1 expression, consequently leading to a reduction of the ox-LDL-induced stress response. The present results are consistent with Wang et al., in which inhibition of the PI3K/Akt pathway decreased Nrf2 transcriptional activity [39]. These findings indicate that ginkgolide B inhibits LOX-1 expression by blocking Akt phosphorylation and increasing Sirt1 expression, which might be an important protective mechanism in endothelial cells.

Altogether, the present findings provide evidence that ginkgolide B can protect against endothelial cell injury by reducing LOX-1 and increasing Sirt1. Ginkgolide B may be a potent drug for the prevention of atherosclerosis. However, the mechanism by which ginkgolide B increases Sirt1 expression should be further studied.
